# Ambient Stability of Sodium-Doped Copper Oxide Obtained through Thermal Oxidation

**DOI:** 10.3390/ma17194823

**Published:** 2024-09-30

**Authors:** Katarzyna Gawlińska-Nęcek, Robert P. Socha, Zbigniew Starowicz, Łukasz Major, Piotr Panek

**Affiliations:** 1 Institute of Metallurgy and Materials Science, Polish Academy of Sciences, Reymonta 25, 30-059 Krakow, Poland; z.starowicz@imim.pl (Z.S.); l.major@imim.pl (Ł.M.); p.panek@imim.pl (P.P.); 2 CBRTP SA Research and Development Center of Technology for Industry, Ludwika Waryńskiego 3A, 00-645 Warszawa, Poland; 3 Jerzy Haber Institute of Catalysis and Surface Chemistry, Polish Academy of Sciences, Niezapominajek 8, 30-239 Krakow, Poland

**Keywords:** copper oxide, thermal oxidation, sodium dopant, stability, heterojunction solar cell

## Abstract

The ambient stability of copper oxide layers produced through thermal oxidation is a critical factor for their application in advanced photovoltaic devices. This study investigates the long-term stability of thermally grown sodium-doped copper oxides fabricated at 300 °C, 500 °C, and 700 °C. The structural, optical, and electronic properties of these oxide layers were examined after a 30-day period to understand how thermal oxidation temperature and sodium doping influence the durability and properties of copper oxide films. The results indicate that the stability of thermal copper oxide increases with oxidation temperature. The film produced at 700 °C maintained consistent optical properties, work function value, and structural integrity over time, demonstrating their robustness against environmental degradation. In contrast, the layers produced at lower temperatures (300 °C and 500 °C) showed more significant changes due to continued oxidation and adsorption from ambient.

## 1. Introduction

In recent years, copper oxide (CuO_x_) has gained significant attention in the field of photovoltaics, particularly as an absorber material in heterojunction solar cells [1,2,3,4] and as a hole transport layer in perovskite solar cells [5,6,7]. This is largely due to its abundance, non-toxicity, and favorable band gap, making it a promising candidate for cost-effective and sustainable solar devices. Among the various forms of copper oxide, cuprous oxide (Cu_2_O) and cupric oxide (CuO) are the most commonly studied as absorbers in heterojunction solar cells [2,3,4]. The synthesis of copper oxides typically involves methods such as chemical vapor deposition (CVD) [8], magnetron sputtering [9], atomic layer deposition (ALD) [10,11,12], spray pyrolysis [13,14], and sol–gel techniques [15,16]. Each method offers specific advantages regarding control over film thickness, uniformity, and composition. However, thermal oxidation of copper substrates remains one of the most straightforward and scalable approaches for producing CuO_x_ layers. This process involves heating copper in an oxygen environment, resulting in the formation of an oxide layer on the surface. The oxidation process can be finely tuned by adjusting parameters such as temperature, oxygen pressure, and duration.

The formation of Cu_2_O and CuO during the thermal oxidation of copper sheets is well-described, as detailed in the study by Valladares et al. [17]. This process involves a sequence of oxidation reactions that are temperature-dependent and influence the final composition and structure of the oxide layer. At lower oxidation temperatures, typically below 400 °C, the oxidation of copper primarily results in the formation of cuprous oxide (Cu_2_O), which is a p-type semiconductor with band gap energy of about 2.1 eV. In this initial stage, oxygen atoms react with copper atoms. The oxide layer grows through the inward diffusion of oxygen and the outward diffusion of copper ions. As the oxidation temperature increases above 400 °C, the process continues, leading to the transition from Cu_2_O to CuO. At intermediate temperatures, a mixed-phase layer is formed, where both Cu_2_O and CuO coexist [17].

In our previous studies, we investigated low-temperature copper oxides produced through the thermal oxidation of copper sheets [18,19]. We highlighted the challenge of stress development caused by the lattice mismatch between the copper substrate and the copper oxide layer, which often led to the delamination of the oxide coating. We emphasized the necessity of proper preparation and polishing of the copper substrate, identifying 300 °C as the optimal oxidation temperature. This temperature allowed for the formation of a uniform and stable oxide layer composed mainly of Cu_2_O with a small amount of CuO [18]. In other work, we demonstrated that the introduction of sodium as a dopant, applied to the surface of a copper sheet immediately before the oxidation process, enables the production of a high-temperature copper oxide layer that is not only low in resistivity but also stable, crack-free, and adherent [20]. Such sodium-doped copper oxide holds significant potential for application in heterojunction solar cells due to its high light absorption and low resistivity. However, it is important to highlight the significant challenges associated with using copper oxide as an absorber in these cells, particularly the difficulty in finding a suitable n-type semiconductor layer and the inherent instability of copper oxide films. Although Minami et al. demonstrated that utilizing n-type oxides such as zinc oxide [21], aluminum-doped zinc oxide [22], gallium oxide [23], aluminum gallium oxide [4], or germanium-doped zinc oxide [24] can result in copper oxide-based solar cells with an efficiency of about 8%, the theoretical efficiency can exceed 20% [25].

Therefore, in this study, we thoroughly examine the long-term stability of thermally produced sodium-doped copper oxides manufactured at 300 °C, 500 °C, and 700 °C. We investigated the structural, optical, and electronic properties of these oxide layers over a 30-day period, which was chosen as sufficient to capture early-stage degradation mechanisms or stability trends under standard environmental conditions, such as air exposure, temperature fluctuations, and humidity. This timeframe also aligns with the practical requirements for assessing the material’s stability during key stages in the production, storage, and transport of solar cells before further layers are applied in the device fabrication process. It provides an early indication of how the copper oxide layers will behave when exposed to real-world conditions, allowing us to identify any significant surface or structural changes that could impact their performance in solar cells. The samples were stored under ambient laboratory conditions, with a temperature of approximately 22 °C, relative humidity of 40–50%, and atmospheric pressure. We aim to provide a comprehensive understanding of how thermal oxidation temperature influences the durability and properties of copper oxide films.

## 2. Materials and Methods

The production processes of the Na-doped copper oxide layer (Na:CuO_x_) by thermal oxidation were carried out using an IR belt furnace under ambient conditions. The samples were heated from the top, and the belt speed was 50 cm/min. The lengths of the zones were as follows: 38 cm, 19 cm, and 19 cm. We used an ETP R220 copper sheet with a thickness of 200 µm as the base material. The source of the Na dopant was the EK1 solution of 5.6% sodium concentration (patent application P.439040), applied to the chemically prepared ETP R220 copper sheet substrates via screen printing. After drying at 80 °C for 20 s, the samples were heated in the IR belt furnace at temperatures of 300 °C, 500 °C, and 700 °C. The produced layers exhibit unique adhesive properties and do not detach from the substrate when stored at room temperature.

The manufactured layers were optically characterized by measuring the reflection coefficient using a UV-VIS-NIR Perkin Elmer Lambda 950 S spectrophotometer (Waltham, MA, USA) equipped with a 150 mm PbS integrating sphere. The layer morphology was examined using transmission electron microscopy (TEM) with a Tecnai F20 200 kV (FEG) microscope (FEI Company, Eindhoven, The Netherlands) equipped with an EDAX energy dispersive X-ray spectroscopy (EDS) detector (Ametek, Mahwah, NJ, USA). Thin foils for TEM investigations were prepared using the focused ion beam (FIB) technique, utilizing a Quanta 200 3D Dual Beam (FIB/SEM) system (FEI Company, Eindhoven, The Netherlands) equipped with an OmniProbe (Oxford Instruments, High Wycombe, UK) micromanipulator. Surface composition was determined using X-ray photoelectron spectroscopy (XPS) in an ultra-high vacuum (1 × 10^−9^ mbar) with a Prevac XPS spectrometer (Rogów, Poland). The work function was calculated based on the Contact Potential Difference (CPD) measured using a single-point Kelvin probe (Photon Institute, Krakow, Poland). The copper oxide morphology was investigated by a Hitachi TM3030 scanning electron microscope with 10 kV of accelerating voltage. The compositional profile of Na:CuO_x__700 °C was determined by SIMS (Secondary Ions Mass Spectrometry) measurement performed using a system equipped with a CAMECA IMS6F magnetic sector instrument. The XRD was performed using Bruker D8 ADVANCE X-ray diffractometer with filtered radiation Cu Kα (λ = 0.154056 nm) at room temperature.

## 3. Results and Discussion

**Figure 1:**
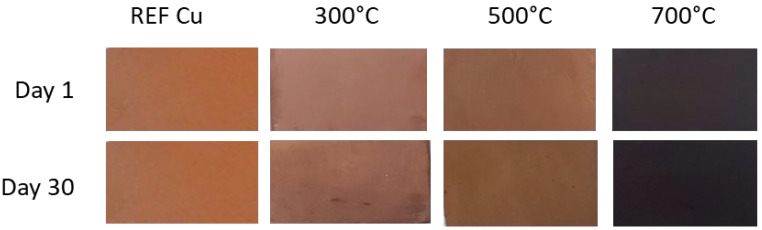
Digital photo of manufactured thermal copper oxide films with sodium dopant on day 1 and after 30 days of storage.

The visual analysis of the sodium-doped copper oxide layers produced at 300 °C, 500 °C, and 700 °C reveals distinct differences in sample coloration (Figure 1). The copper substrate oxidized at 300 °C (Na:CuO_x__300 °C) developed a slightly milky appearance, indicating significant light scattering, which is characteristic of a fine-grained or porous surface structure. This suggests that the layer is likely composed predominantly of Cu_2_O, which, when thin, exhibits a yellowish tint. When the oxidation temperature was increased to 500 °C (Na:CuO_x__500 °C), the resulting layer displayed a brown color. The more intense coloration compared to Na:CuO_x__300 °C suggests a thicker or more uniform Cu_2_O coating with some CuO share. At 700 °C (Na:CuO_x__700 °C), the copper oxide film appeared black, indicative of the formation of CuO and a high degree of light absorption.

After 30 days of exposure to air, the samples produced at 300 °C and 500 °C darkened, suggesting further oxidation or surface changes over time. In contrast, Na:CuO_x__700 °C maintained its black color with no noticeable changes, showing that the high-temperature oxide layer is more stable and resistant to further environmental degradation. The XRD results for the obtained layers are provided in the Supplementary Materials (Figure S1). Due to the surface nature of the observed changes, the XRD patterns did not show any significant differences in the bulk of the layers measured immediately after preparation and after 30 days of storage.

**Figure 2:**
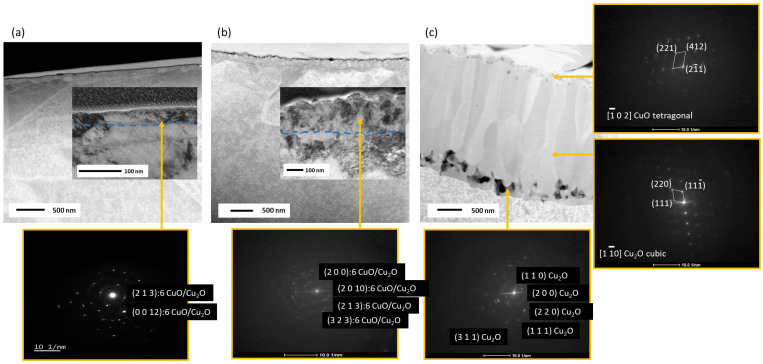
STEM and TEM cross-section with diffraction pattern of sodium-doped copper oxide manufactured at (**a**) 300 °C and (**b**) 500 °C and (**c**) STEM cross-section with diffraction patterns of sodium-doped copper oxide manufactured at 700 °C.

The cross-sections of thermally produced copper oxide layers on Cu sheets with the surface modified by Na are presented in Figure 2. The images include STEM pictures for all samples and TEM BF cross-sections for the Na:CuO_x_ produced at 300 °C and 500 °C. Diffraction patterns for these layers are also shown. The investigation was conducted only on freshly prepared samples. The STEM and TEM analysis reveals that the Na:CuO_x_ formed at 300 °C consists of fine crystals, as indicated by the ring-like diffraction pattern, which is a result of homogeneous nucleation throughout the oxide film due to lower temperature and slower diffusion rate. The thickness of the coating is approximately 40 nm. It should also be indicated that the layer adheres well to the Cu substrate, with no visible holes at the Cu/Na:CuO_x_ interface. Similarly, the Na:CuO_x_ produced at 500 °C also comprises fine crystals with a thickness of around 200 nm. Higher oxidation temperature and increased diffusion rate allow for faster layer growth while maintaining the fine-grain structure. The Na:CuO_x__500 °C also shows good adhesion to the substrate without any gaps at the interface.

In contrast, the film formed at 700 °C exhibits a more complex structure. Near the Cu substrate, the 270 nm layer consists of fine, equiaxed crystals of Cu_2_O. This is followed by a zone of Cu_2_O columnar crystals with a thickness of about 2.2 µm. The steep thermal gradient and enhanced diffusion rates favor the elongation of these grains. The thermal gradient occurs in a belt furnace where copper substrates are applied to one side of the heating. That causes a directional heat flow from the hot side to the cooler side, namely from the substrate’s surface that is directly exposed to the heat source, forming a hot zone, while the bulk material remains relatively cooler. The final surface region of the layer consists of fine CuO crystals. The thickness of this film is about 170 nm. Notably, there are visible holes in the coating at the interface with the Cu substrate. The reason for this might be due to localized stresses and differences in thermal expansion between the Cu substrate and the growing oxide film, leading to void formation. Despite this, the oxide layer remained stable and did not detach. It is important to highlight that the sodium-doped copper oxide layers were prepared using a precursor solution with a sodium concentration of 5.6%, and all samples were coated with the same initial doping film, as the oxidation kinetics were not fully understood at the time. Interestingly, neither the cross-sectional STEM images (Figure 2) nor the surface SEM analysis (Figure S2) showed any sodium residues, which could indicate an excess of the dopant. This suggests that the sodium dopant was effectively incorporated into the oxide layer. It is hypothesized that the dopant concentration decreases as the oxidation temperature increases due to the oxide film thickening. SIMS analysis (Figure S3) of Na:CuO_x__700 °C, chosen for its superior stability, revealed a sodium concentration of approximately 10^17^ atoms/cm^3^. Sodium doping plays a crucial role in preventing the oxide layer from delaminating from the metallic substrate, even when subjected to high temperatures. This is a critical improvement, as we previously described delamination issues in our earlier work under similar thermal conditions without doping [18]. The sodium appears to reduce the stress at the oxide–substrate interface, enabling better adhesion and stability of the layer.

**Figure 3:**
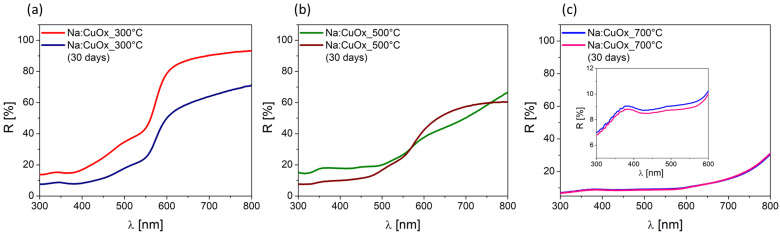
Reflection coefficient of manufactured Na:CuO_x_ layers manufactured at (**a**) 300 °C, (**b**) 500 °C, and (**c**) 700 °C, measured immediately after processing and after 30 days of storage.

The optical properties were evaluated by measuring the reflectance (R%) and diffuse reflectance (R_diff_%) in the wavelength range of 300–800 nm (Figure 3). The R% value refers to the total reflectance, which includes both specular and diffuse components. The diffuse reflectance (R_diff_%) corresponds to the part of reflected light that is scattered in many directions after interacting with a rough surface, as opposed to specular reflectance, which is the mirror-like reflection where light is reflected at a single angle. Measurements were conducted using a Perkin Elmer UV-VIS-NIR Lambda 950 S spectrophotometer equipped with an integrating sphere, which collects light reflected from the sample in all directions, enabling the measurement of total reflectance. The integrating sphere’s removable cover allows the specular component to escape while the remaining diffuse light is collected by the detector, isolating the diffuse component. The analysis was performed both on the day of sample fabrication and after 30 days of storage.

On the first day, the Na:CuO_x_ produced at 300 °C showed the highest reflection coefficients, with values of 15% at 370 nm (R370) and 44% at 550 nm (R550). This indicates a relatively high reflectivity in both the UV and visible regions, suggesting a less absorbing and more reflective behavior. The layer produced at 500 °C showed lower reflection coefficients across the measured range, with 18% of R370 and 27% of R550. Although the reflectivity is lower than that of the Na:CuO_x__300 °C sample, it still indicates a moderate level of absorption. Finally, the copper oxide film produced at 700 °C had the lowest reflection coefficients, with R370 and R550 both at 9%. This is attributed to the nearly complete absorption of light by this layer, which appears black. The high absorption suggests a thicker and more uniform oxide layer with enhanced light-trapping properties. To facilitate comparison, the reflection coefficients for wavelengths of 370 nm and 550 nm are summarized in Table 1.

After 30 days of storage, significant changes in the R% values were observed for Na:CuO_x__300 °C and Na:CuO_x__500 °C, while Na:CuO_x__700 °C remained stable. It was found that the reflectance of Na:CuO_x__300 °C reduced to 8% for R370 and to 25% for R550. For copper oxide produced at 500 °C, the R370 and R550 lowered to 9% and 25%, respectively. What is more, for Na:CuO_x__500 °C, the formation of a better-defined reflection edge was observed, suggesting some degree of structural stabilization over time. The noticed decrease in reflectivity for both Na:CuO_x__300 °C and Na:CuO_x__500 °C can be attributed to further oxidation of the copper oxide layer or to the adsorption of moisture and other atmospheric contaminants on the samples’ surface.

The stability of Na:CuO_x__700 °C suggests that the higher temperature oxidation process produces a more stable and uniform oxide layer. The consistent optical properties indicate minimal changes in the surface morphology and composition, making it more resistant to environmental factors.

The band gap energy (Eg) of sodium-doped copper oxide layers was calculated using the Kubelka–Munk method, which involves converting the diffuse reflectance data into an absorption coefficient by the following formula:

(1) $$F(R_{\mathrm{diff}})=\frac{{\left(1-R_{diff}\right)}^{2}}{{2R}_{diff}}$$

**Figure 4:**
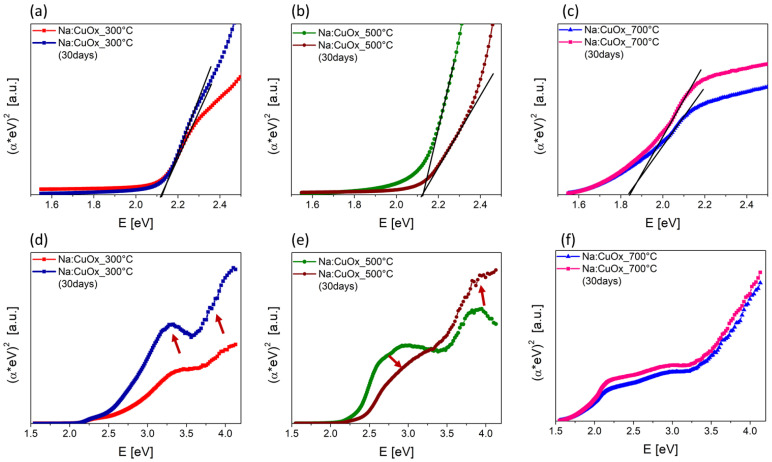
Kubelka–Munk plots for sodium-doped copper oxide produced at (**a**,**d**) 300 °C, (**b**,**e**) 500 °C, and (**c**,**f**) 700 °C.

The extrapolation of the linear portion of the (F(R_diff_)⋅hν)^n^ versus hν, where n depends on the type of electronic transition (for directly allowed transitions, n = 2), to the photon energy axis (hν) determined the Eg (Figure 4).

The band gap energies were found to be 2.12 eV for both Na:CuO_x__300 °C (Figure 4a) and Na:CuO_x__500 °C (Figure 4a), which is very close to the theoretical Eg for pure Cu_2_O (2.1 eV). The Eg for Na:CuO_x__700 °C was 1.84 eV, which may correspond to a transition to CuO. Surprisingly, after 30 days of storage in ambient conditions, the estimated Eg values for the Na-doped copper oxide films remained stable for all samples. However, changes were observed in the deeper bands, as indicated by red arrows in Figure 4d,e. For the film produced at 300 °C, the bands at 4.0 eV and 3.2 eV became more pronounced, which is characteristic of Cu_2_O. This implies that the layer produced at 300 °C may be a non-stoichiometric oxide as prepared. For the film produced at 500 °C, the transition over time resulted in a shape more closely resembling that of CuO, similar to Na:CuO_x__700 °C. The middle shoulder became weaker, while the one around 4.0 eV increased. This may signal structural rearrangements of Cu_2_O during storage or even the emergence of a CuO phase, likely due to ongoing surface oxidation. Finally, the sample produced at 700 °C did not reveal any significant changes, confirming its stability.

**Figure 5:**
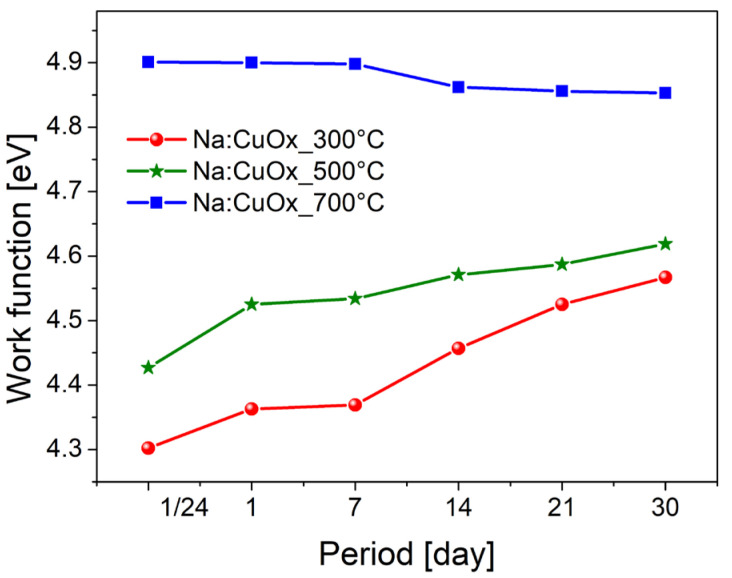
Change in work function of the Na:CuO_x_ obtained at 300 °C, 500 °C, and 700 °C over time.

The work function (ϕ) of sodium-doped copper oxide thin films was determined through measurements of the Contact Potential Difference (CPD) using a Kelvin probe. Prior to the investigation, the reference Au electrode was calibrated with Highly Ordered Pyrolytic Graphite (HOPG) to ensure accuracy. The evolution of the work function over time was closely monitored and is presented in Figure 5. Measurements were taken immediately after the sample preparation, as well as at intervals of 1, 7, 14, 21, and 30 days post-production. The results indicate a clear correlation between the oxidation temperature and the initial work function of the thin films. Specifically, the work function values increase with the oxidation temperature, measuring 4.3 eV for Na:CuO_x__300 °C, 4.42 eV for Na:CuO_x__500 °C, and 4.9 eV for Na:CuO_x__700 °C. Over time, the work function of the copper oxide layers produced at 300 °C and 500 °C presented a gradual rise, reaching 4.55 eV and 4.6 eV, respectively, after 30 days. This increase might imply ongoing surface changes, possibly due to further oxidation or adsorption of atmospheric contaminants, which can alter the surface electronic properties that correspond to the optical analysis results. The work function of the layer fabricated at 700 °C remained stable for the first 7 days, then a slight decrease to 4.85 eV was observed.

**Figure 6:**
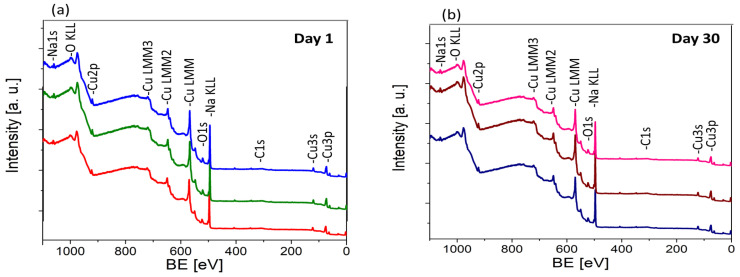
XPS survey spectra of sodium-doped copper oxide manufactured at 300 °C (red/navy blue), 500 °C (green/brown), and 700 °C (blue/pink) measured at (**a**) day 1, and (**b**) day 30.

The definitive investigation that sheds light on the surface processes responsible for the stability of the produced oxides was conducted using XPS measurement. Figure 6 presents the XPS survey spectra of the copper oxide samples analyzed immediately after fabrication (Figure 6a) and after 30 days of storage in ambient air (Figure 6b). The surface composition determined by fitting XPS spectra is collected in Table 2.

The analysis reveals several trends regarding the surface composition of the films as a function of the oxidation temperature. Firstly, it was observed that as the oxidation temperature increases, the concentration of copper (Cu) on the film surface rises while the concentration of sodium (Na) decreases. This trend suggests that higher temperatures promote more effective oxidation of the copper substrate, leading to a higher relative abundance of copper on the surface. Simultaneously, the decrease in surface sodium concentration at higher temperatures may signal its uniform distribution in bulk oxide with higher thicknesses. Additionally, the data show a significant reduction in carbon (C) with increasing oxidation temperature, which is attributed to the more complete removal of residual organics, resulting in a cleaner surface.

After one month of exposure to air, XPS analysis revealed a rise in the organic content on the surface of all samples. This adsorption appears to be more pronounced on sodium-rich regions of the surface, as evidenced by the concurrent decrease in surface sodium concentration. For the sample produced at 700 °C, the lower detection of sodium on the surface, as well as the smallest carbon content increase after storage, supports the notion of effective sodium incorporation and diffusion throughout the bulk of the oxide layer. This efficient embedding of sodium within the oxide matrix could contribute to the enhanced stability and uniformity of the film, as observed in other characterization techniques.

**Figure 7:**
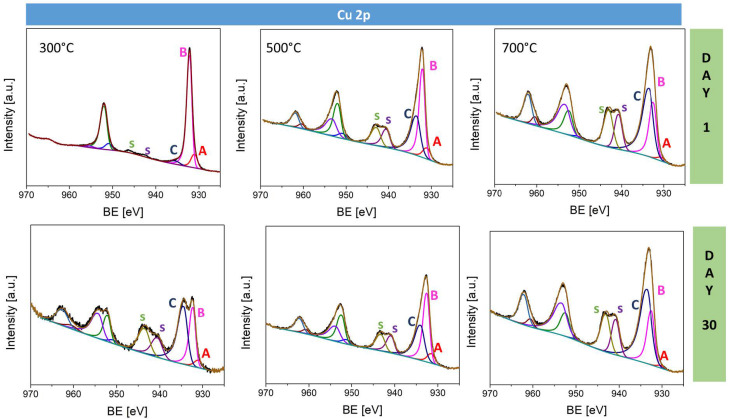
High-resolution Cu 2p spectra for sodium-doped copper oxide samples fabricated at 300 °C, 500 °C, and 700 °C measured on day 1 and after 30 days of storage.

High-resolution spectra of Cu 2p, O 1s, C 1s, and Na 1s core excitations were deconvoluted to reveal the contributions of various chemical species. The Cu 2p spectra (Figure 7) were fitted with five doublet components that can be assigned to specific electronic states, namely Cu–Cu (metallic copper) at 931 eV, Cu^+^–O at 932.0 eV, Cu^2+^–OH or Cu^2+^–O (CuO or other copper (II) species) at 935.4 eV, and two shake-up satellites corresponding to π* electron excitation, typically associated with Cu^2+^ (Table 3). For the sample oxidized at 300 °C, the surface predominantly consists of Cu_2_O, as indicated by the strong peak at 932 eV, with a minor contribution from metallic copper at 931 eV. The presence of very weak shake-up satellites (labeled s) further confirms that the surface is primarily composed of Cu or Cu_2_O, as these species typically exhibit minimal satellite intensity. The Na:CuO_x__500 °C surface consists of approximately 51.7% Cu_2_O and 40% CuO. This change is reflected in the increased intensity of the peak at 935.4 eV (component C), implying a higher proportion of CuO. For the sample oxidized at 700 °C, CuO was found as the predominant phase (62.8%) of the surface composition. This is evidenced by the prominent satellite features and the strong peak at 935.4 eV. After 30 days of storage in air, the Na:CuO_x__300 °C sample shows significant changes. Notably, there is a marked increase in the intensity of the satellites. The position of Cu 2p_3/2_ moved from 932.14 eV to 932.5 eV, and another peak at 934.5 appeared, pointing out an increased amount of CuO on the surface. Quantitative analysis shows that the surface composition has changed to 36.9% Cu_2_O and 57.6% CuO. Na:CuO_x__500 °C exhibits relatively stable spectra after 30 days; however, the shift in Cu 2p_3/2_ position to higher binding energy, from 932.19 eV to 932.68 eV, suggests further oxidation to CuO. Finally, no significant changes in the intensity and the position of the Cu 2p peaks for Na:CuO_x__700 °C were observed. This stability can indicate that a copper oxide thin film obtained at 700 °C is more resistant to further oxidation.

**Figure 8:**
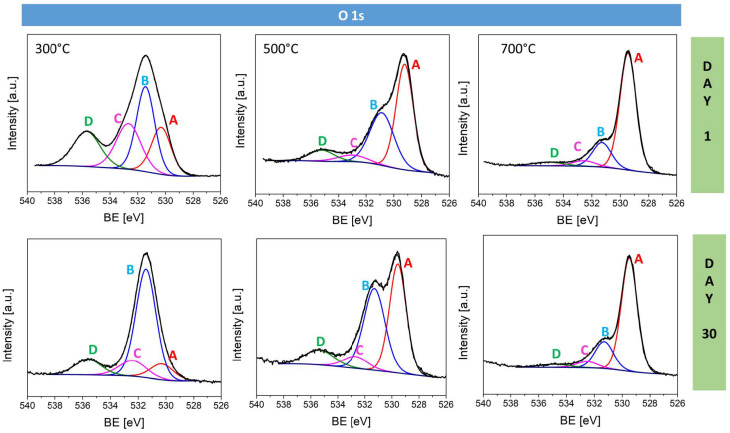
High-resolution O 1s spectra for sodium-doped copper oxide samples fabricated at 300 °C, 500 °C, and 700 °C measured on day 1 and after 30 days of storage.

The components of the O 1s (Figure 8) spectra can be assigned to oxygen bonded to metal at 530.3 eV, hydroxyl groups at 531.3 eV, adsorbed water at 532.7 eV, and organic carbonyl groups at 535.7 eV (Table 4). The analysis revealed that as the oxidation temperature increases, there is a noticeable rise in the O-Metal component at 530.3 eV, indicating a higher proportion of metal–oxygen bonds, consistent with more extensive oxidation at higher temperatures. Concurrently, the number of O=C bonds decreases with temperature, reflecting the more effective removal of residual organic at elevated temperatures. After 30 days of storage, the number of O-Metal bonds significantly decreases in both the Na:CuO_x__300 °C and Na:CuO_x__500 °C films while the number of hydroxyl groups increases. For Na:CuO_x__300 °C, this is evident as a narrowing of the O 1s peak, while for the Na:CuO_x__500 °C sample, it is observed as a raised left shoulder of the peak at 531.3 eV. The copper oxide produced at 700 °C is more stable, showing minimal change in the O 1s spectrum after 30 days. It also has less organic adsorbate compared to the films produced at 300 °C and 500 °C.

**Figure 9:**
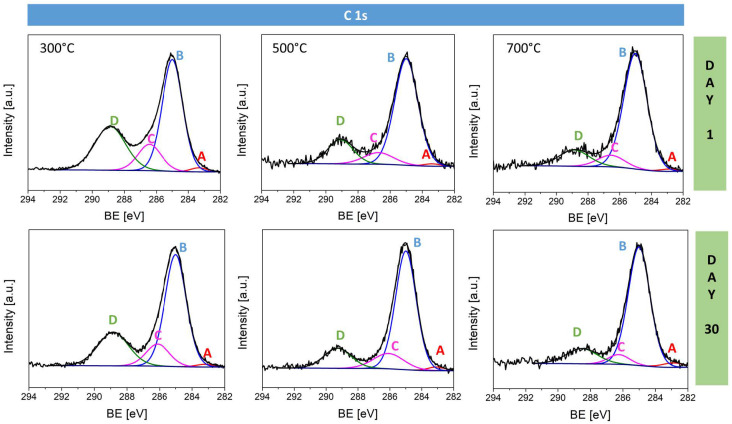
High-resolution C 1s spectra for sodium-doped copper oxide samples fabricated at 300 °C, 500 °C, and 700 °C measured on day 1 and after 30 days of storage.

The C 1s spectra (Figure 9) were deconvoluted with four components: carbon–metal at 283.3 eV, aliphatic hydrocarbons, typical of adsorbed organic contaminants at 285 eV, alcohol groups at 286.4 eV, and carboxyl groups at 288.9 eV (Table 5). It was found that the number of carbon–metal bonds reduce with temperature growth, again likely due to the more effective oxidation and removal of organic adsorbate at higher temperatures. Moreover, the number of C–OH and COOH bonds decreases with the manufacturing temperature due to a more complete removal of these groups during the oxidation process. After 30 days of storage, the number of carbon–metal bonds rises for all samples as a result of the re-adsorption of carbon species from the environment. The C–C component also increases, while the number of C–OH and COOH decreases, suggesting ongoing surface reactions or the adsorption of organic contaminants over time.

**Figure 10:**
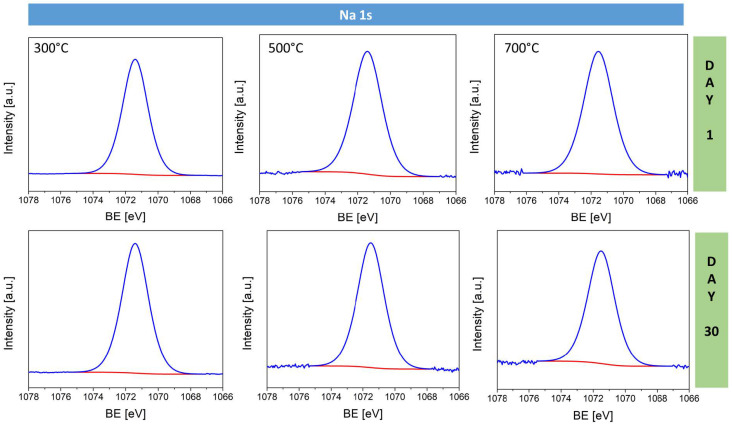
High-resolution Na 1s spectra for sodium-doped copper oxide samples fabricated at 300 °C, 500 °C, and 700 °C measured on day 1 and after 30 days of storage.

The Na 1s spectra (Figure 10) show a single component at 1071.4 eV corresponding to Na^+^–O, similar to those found in Na_2_O. There is no clear impact of the processing conditions on the sodium chemical state, as this peak remains consistent across all samples and processing conditions. However, the overall intensity of the sodium peak grows with increasing oxidation temperature, which confirms that sodium is more effectively incorporated into the bulk of the oxide layer rather than remaining on the surface.

## 4. Discussion and Conclusions

This study investigated the long-term stability of sodium-doped copper oxide layers produced via thermal oxidation at temperatures 300 °C, 500 °C, and 700 °C. The findings underscore the significant impact of oxidation temperature on the physical and electronic properties of the oxide layers. Cross-sectional analysis confirmed that the layer thickness increases with temperature, accompanied by notable changes in morphology. The copper oxide layers produced at 300 °C and 500 °C exhibited fine crystalline structures. In comparison, the layer produced at 700 °C showed columnar crystals of Cu_2_O with equiaxed grains at the top (CuO) and bottom (Cu_2_O) of the layer. The film produced at 700 °C demonstrated remarkable stability, maintaining consistent optical properties and work function values over a 30-day storage period. In contrast, the layers produced at 300 °C and 500 °C showed significant changes in reflectivity and work function over time, indicating the adsorption of water and carbon species on the surface and ongoing oxidation. The reflection coefficients for Na:CuO_x__300 °C and Na:CuO_x__500 °C decreased over time, while for the Na:CuO_x__700 °C layer, they remained stable. The band gap energy did not change for all samples; however, for Na:CuO_x__300 °C and Na:CuO_x__500 °C, deeper band changes were confirmed, which suggests further oxidation of these layers in air. The work function of the freshly prepared Na:CuO_x__700 °C achieved the highest value of 4.9 eV, which remained stable over the observation period. This differs from the films fabricated at 300 °C and 500 °C, which exhibited increases in work function from 4.3 eV to 4.55 eV for Na:CuO_x__300 °C and from 4.42 eV to 4.6 eV for Na:CuO_x__500 °C. XPS analysis confirmed that the surface of freshly prepared Na:CuO_x__300 °C is primarily composed of Cu_2_O, Na:CuO_x__500 °C is formed of 51.7% Cu_2_O and 40% CuO, while Na:CuO_x__700 °C consists of 34.9% of Cu_2_O and 62.8% CuO. The presence of organic residues was detected on all samples, with amounts decreasing as the oxidation temperature increased. Additionally, the sodium content on the oxide surfaces decreased with oxidation temperature, pointing out more efficient dopant distribution within the Na:CuO_x__700 °C layer. After 30 days of storage, additional CuO was detected on the Na:CuO_x__300 °C surface, which is evidenced by the appearance of satellites, the shift of the main Cu 2p_3/2_ peak, and the emergence of an additional peak at 934.5 eV in Cu 2p spectrum. A similar position shift of the Cu 2p_3/2_ peak toward higher binding energies was found for Na:CuO_x__500 °C, indicating further oxidation. In contrast, the copper oxide layer produced at 700 °C was more anchored, with no further oxidation in air. These observations correspond to the optical results collected after 30 days. The organic species and water were adsorbed from the environment on all samples during storage; however, the most significant increase was observed for Na:CuO_x__300 °C, while the smallest one was found for Na:CuO_x__700 °C, confirming superior ambient stability.

The study demonstrates that sodium doping, combined with high-temperature thermal oxidation at 700 °C, is a promising approach for producing stable and efficient copper oxide layers suitable for advanced photovoltaic applications.

## Figures and Tables

**Table 1:** The reflection coefficients at 370 nm and 550 nm.

	R370 (%)	R550 (%)	R370 (30 Days) (%)	R550 (30 Days) (%)
Na:CuO_x__300 °C	15	44	8	25
Na:CuO_x__500 °C	18	27	9	25
Na:CuO_x__700 °C	9	9	9	9

**Table 2:** Surface composition (atomic %) determined by fitting XPS spectra for all analyzed samples of copper oxide on day 1 and day 30.

	Day 1				Day 30			
Excitation	C1s	O1s	Cu2p	Na1s	C1s	O1s	Cu2p	Na1s
Binding energy [eV]	285	529	933	1071	285	529	933	1071
Na:CuO_x__300 °C	31.3	40.5	12.2	16.1	41.6	38.4	11.8	8.2
Na:CuO_x__500 °C	14.6	31.9	38.2	15.3	28.2	32.5	30.7	8.6
Na:CuO_x__700 °C	14.2	33.0	45.3	7.4	18.3	31.4	45.1	5.3

**Table 3:** Surface composition (atomic %) determined by fitting Cu 2p spectra for all analyzed samples of copper oxide on day 1 and day 30.

	Day 1			Day 30		
Spectrum Component	A	B	C	A	B	C
Binding energy [eV]	931.0	932.0	935.4	931.0	932.0	935.4
Type of bonding	Cu–Cu	Cu^+^–O	Cu^2+^–OH, Cu^2+^–O	Cu–Cu	Cu^+^–O	Cu^2+^–OH, Cu^2+^–O
Na:CuO_x__300 °C	12.0	83.7	4.3	5.6	36.9	57.6
Na:CuO_x__500 °C	8.3	51.7	40.0	8.2	50.8	41.0
Na:CuO_x__700 °C	2.3	34.9	62.8	1.4	31.6	67.0

**Table 4:** Surface composition (atomic %) determined by fitting O1s spectra for all analyzed samples of copper oxide on day 1 and day 30.

	Day 1				Day 30			
Spectrum Component	A	B	C	D	A	B	C	D
Binding energy [eV]	530.3	531.3	532.7	535.7	530.3	531.3	532.7	535.7
Type of bonding	O–metal	–OH	H_2_O	O=C	O–metal	–OH	H_2_O	O=C
Na:CuO_x__300 °C	20.4	34.1	24.8	20.7	10.8	64.3	12.7	12.2
Na:CuO_x__500 °C	51.1	35.7	5.5	7.7	42.6	40.4	6.7	10.2
Na:CuO_x__700 °C	73.1	17.6	5.0	4.3	71.5	19.6	5.2	3.7

**Table 5:** Surface composition (atomic %) determined by fitting C1s spectra for all analyzed samples of copper oxide on day 1 and day 30.

	Day 1				Day 30			
Spectrum Component	A	B	C	D	A	B	C	D
Binding energy [eV]	283.3	285.0	286.4	288.9	283.3	285.0	286.4	288.9
Type of bonding	C–metal	–C–C	–C–OH	–COOH	C–metal	–C–C	–C–OH	–COOH
Na:CuO_x__300 °C	1.5	53.1	14.2	31.2	1.3	60.0	13.0	25.8
Na:CuO_x__500 °C	1.2	71.2	10.0	17.6	1.8	69.5	13.2	15.5
Na:CuO_x__700 °C	1.1	76.1	8.8	13.9	3.0	76.8	6.2	14.0

## Data Availability

Data are contained within the article or Supplementary Materials.

## Supplementary Materials

The following supporting information can be downloaded at: https://www.mdpi.com/article/10.3390/ma17194823/s1, Figure S1: XRD pattern of sodium-doped copper oxide produced at 300 °C, 500 °C, and 700 °C; Figure S2: SEM morphology of freshly prepared and 30-day storage sodium-doped thermal copper oxide manufactured at 300 °C, 500 °C, and 700 °C; Figure S3: SIMS depth profile of sodium-doped copper oxide produced at 700 °C.
