# Supplementary material for: Ambient Stability of Sodium-Doped Copper Oxide Obtained through Thermal Oxidation
Source: Materials (Basel). 2024 Sep 30;17(19):4823. doi: 10.3390/ma17194823 (PMC11478185; doi:10.3390/ma17194823)
Supplement: Supplementary file 1 [file materials-17-04823-s001.zip › materials-3209394-supplementary.pdf]

*Supporting Information*

# Ambient Stability of Sodium-Doped Copper Oxide Obtained through Thermal Oxidation

**Katarzyna Gawlińska-Nęcek [1,*], Robert P. Socha [2,3,*], Zbigniew Starowicz [1], Łukasz Major [1] and Piotr Panek [1]**

1 Institute of Metallurgy and Materials Science, Polish Academy of Sciences, Reymonta 25, 30-059 Krakow, Poland; z.starowicz@imim.pl (Z.S.); l.major@imim.pl (Ł.M.); p.panek@imim.pl (P.P.)
2 CBRTP SA Research and Development Center of Technology for Industry, Ludwika Waryńskiego 3A, 00-645 Warszawa, Poland
3 Jerzy Haber Institute of Catalysis and Surface Chemistry, Polish Academy of Sciences, Niezapominajek 8, 30-239 Krakow, Poland
* Correspondence: k.gawlinska@imim.pl (K.G.-N.); robert.socha@cbrtp.pl (R.P.S.)

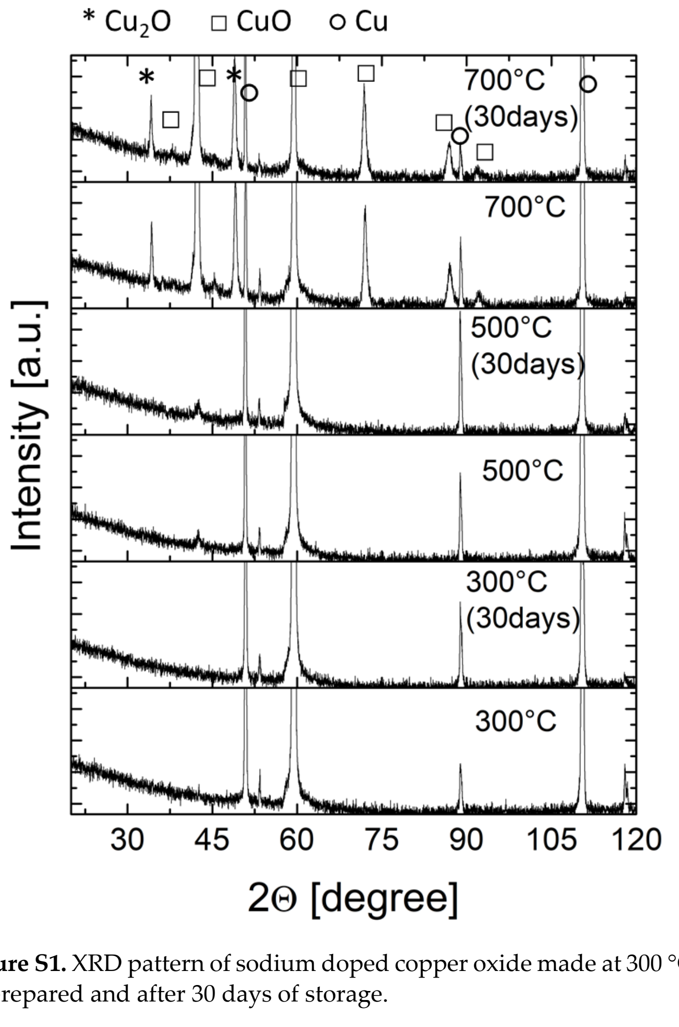


**Figure S1.** XRD pattern of sodium doped copper oxide made at 300 °C, 500 °C, and 700 °C measured as prepared and after 30 days of storage.

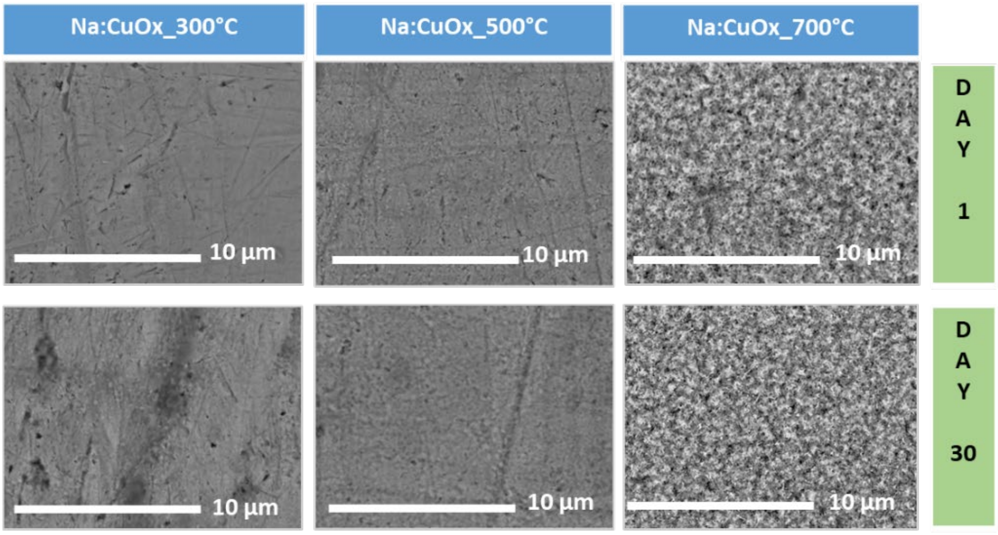


**Figure S2.** SEM morphology of freshly prepared and 30 day storage sodium doped thermal copper oxide manufactured at 300 °C, 500 °C, and 700 °C.

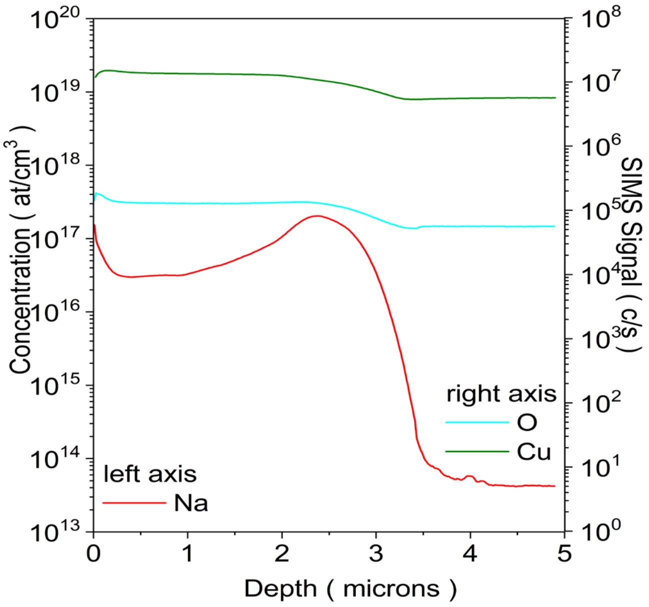


**Figure S3.** SIMS depth profile of sodium doped copper oxide made at 700 °C.
